# Recognition Task-Based Detection Score: A Task-Oriented Evaluation Metric for Infrared Image Colorization

**DOI:** 10.3390/s26061807

**Published:** 2026-03-13

**Authors:** Hao Wang, Jiaming Cai, Yao Hu, Chenglong Zhang, Qun Hao

**Affiliations:** 1 School of Optics and Photonics, Beijing Institute of Technology, Beijing 100081, China; 3120225339@bit.edu.cn (H.W.); 18106955249@163.com (J.C.); 3220250662@bit.edu.cn (C.Z.); qhao@bit.edu.cn (Q.H.); 2 National Key Laboratory on Near-Surface Detection, Beijing 100072, China; 3 School of Optoelectronic Engineering, Changchun University of Science and Technology, Changchun 130022, China

**Keywords:** infrared image colorization, quality evaluation, object detection, task-oriented metric

## Abstract

**Highlights:**

**What are the main findings?**

- A task-oriented colorization evaluation metric (RDS) is proposed that measures colorization quality through object detection performance, incorporating three key design characteristics: position robustness via IoU-based matching, fine-grained interpretability through category-level analysis, and task adjustability through flexible category partitioning strategies.
- Experiments demonstrate that RDS not only maintains consistency with traditional metrics under standard conditions but also exhibits superior stability under registration errors (5.65% improvement vs. 11–70% degradation) and uniquely enables category-level performance diagnosis and flexible evaluation dimension adjustment—capabilities that traditional pixel-based metrics lack.

**What are the implications of the main findings?**

- By providing the evaluation criteria that directly reflect downstream task performance, RDS guides the development of infrared colorization models toward practical applicability rather than pixel-level similarity, driving innovation in model architectures and training strategies for infrared imaging applications.
- The three design characteristics enable RDS to provide reliable evaluation under imperfect registration conditions, reveal category-specific model weaknesses for targeted improvements, and adapt evaluation focus to match diverse application requirements, supporting more effective model optimization in practical deployment scenarios.

**Abstract:**

Infrared image colorization has gained widespread attention in recent years as an important means of enhancing image visibility and semantic expression. However, existing evaluation methods mostly rely on pixel-level differences or feature distribution distances, failing to comprehensively reflect the usability of colorization results in practical tasks. To address this, we propose a task-oriented colorization quality evaluation metric called Recognition-Task based Detection Score (RDS), which uses the recognition accuracy of object detection models on colorized images as a proxy indicator to measure their actual performance in downstream tasks, thereby achieving consistency between image quality assessment and task performance. RDS incorporates three key characteristics in its design: enhancing position robustness through the matching mechanism of object detection tasks, providing fine-grained interpretability through category-level accuracy calculation, and achieving task adjustability through flexible category division strategies. Systematic experiments conducted on both NIR–RGB and FLIR-5C datasets demonstrate that RDS maintains good subjective–objective consistency with traditional metrics under standard registration conditions, exhibits superior stability under registration error scenarios, and possesses fine-grained interpretability and task adjustability that traditional metrics lack. RDS maintains a 5.7% improvement in discriminative Score Gap under misalignment while PSNR degrades by 69.8%, and flexible category merging raises TIC-CGAN’s RDS from 76.05% to 96.45% on unseen scenes, providing more practically valuable criteria for the evaluation and optimization of infrared colorization models.

## 1. Introduction

Infrared imaging technology, independent of visible light illumination, possesses the capability of stable perception in complex environments and is widely applied in scenarios such as night vision surveillance and autonomous driving [1]. However, compared to three-channel visible light images, infrared images are typically single-channel grayscale images with limited degrees of freedom in color space and insufficient semantic information, leading to constrained performance in object detection and recognition [2,3]. To alleviate the information insufficiency of infrared grayscale images, researchers have introduced Image-to-Image Translation technology, which achieves style transformation through mapping relationships and is widely applied in tasks such as image enhancement and style transfer [4,5,6,7]. Prior to the dominance of GAN-based approaches, Convolutional Neural Networks (CNNs) served as the primary deep learning framework for infrared image colorization. Representative works include deep multi-scale CNNs for NIR-to-RGB transfer [8], S-shape network architectures for infrared colorization [9], asymmetric codec-based CNN methods for near-infrared colorization [10], and U-Net-based CNNs for predicting visible spectrum images from near-infrared illumination [11]. In recent years, with the development of Generative Adversarial Networks (GANs) [12,13,14,15], GAN-based image translation methods have become the mainstream approach for grayscale image colorization [8,16,17,18,19,20,21,22,23,24,25,26]. These methods achieve the conversion from infrared grayscale images to color images through deep feature extraction and pixel mapping, significantly enhancing the visual presentation of images. However, methods regarding scientific and objective evaluation of the quality of colorization results remain an urgent problem to be solved.

Evaluation metrics play a crucial driving role in advancing image generation and translation tasks. In the field of image generation, the introduction of Fréchet Inception Distance (FID) [27] directly propelled the transition of generative models from subjective evaluation to objective quantification, catalyzing the rapid iteration of a series of high-quality generative models such as StyleGAN and Diffusion [28]. As Jayasumana et al. [29] pointed out: in many problems of machine learning, reliable evaluation metrics are key to driving progress. Research by Benny et al. also demonstrates that evaluation metrics not only provide unified standards for model comparison but, more importantly, guide research directions and promote innovation in model architectures and training strategies [30].

However, in the field of infrared image colorization, the development of evaluation systems has severely lagged behind model innovation, becoming a bottleneck constraining field advancement. Existing evaluation methods are mainly divided into two categories: no-reference metrics and full-reference metrics. No-reference evaluation metrics primarily evaluate through comparison of images before and after colorization, requiring no reference images. Luo et al. [8] proposed a metric called Average Precision of Canny Edges (APCE) to assess edge consistency before and after thermal infrared grayscale image colorization, but it cannot evaluate the crucial color changes in colorization problems. Full-reference evaluation metrics quantify image quality by comparing pixel-level differences or feature distribution differences between generated images and real reference images. Among them, Structural Similarity Index (SSIM) [31] emphasizes consistency in luminance, contrast, and structural information; Peak Signal-to-Noise Ratio (PSNR) [32] reflects the energy intensity of image reconstruction errors; and Fréchet Inception Distance (FID) [27] evaluates the proximity of image distributions in high-dimensional feature space. However, these metrics all focus on measuring the similarity between generated images and reference images, failing to reflect the support capability of colorization results for downstream tasks in practical applications.

To advance the colorization technology toward practical application, the evaluation system needs to achieve a fundamental transformation: from pixel-level similarity evaluation to task-oriented evaluation. Research by Zhang et al. [33] demonstrates that colorization as a pretraining task can significantly improve object detection and semantic segmentation performance, indicating that the colorization quality should be assessed from its support capability for downstream tasks. Recent studies further confirm that feature representations learned through colorization can be effectively transferred to visual tasks such as detection and segmentation, significantly enhancing downstream task performance [34,35]. This indicates a strong correlation between colorization quality and downstream task performance, and the evaluation system should reflect this task value.

To achieve this transformation, task-oriented evaluation metrics need to possess three key capabilities. First, it requires position robustness to adapt to registration errors. Infrared and color images are often accompanied by non-negligible pixel-level misalignment [20], requiring evaluation methods to tolerate reasonable registration errors and maintain evaluation stability and reliability under imperfect image alignment conditions. Second, it requires providing fine-grained performance analysis capability. Benny et al. [30] pointed out in their research on conditional image generation evaluation that fine-grained category-level evaluation is crucial for identifying model weaknesses and guiding targeted improvements. In colorization tasks, this diagnostic capability can support in-depth analysis and optimization of model performance, enabling researchers to accurately locate performance differences in models across different semantic categories. Third, it requires flexible adjusting evaluation criteria according to task requirements. Infrared image colorization serves two distinct practical goals: color restoration, which requires the model to accurately reproduce the specific colors of known objects in familiar scenes, and structure preservation, which requires the model to maintain semantic structural integrity when generalizing to unseen scenes where color prediction is inherently ambiguous. A practically valuable evaluation system must be able to shift its focus between these two orientations rather than applying a single fixed standard [36]. The evaluation system needs to be able to adjust evaluation criteria according to differences in specific application scenarios, avoiding the use of a single standard to measure diversified task objectives.

In response to the above requirements, this paper proposes Recognition Task-Based Detection Score (RDS), which uses the recognition accuracy of object detection models on colorized images as a proxy indicator for image colorization quality. It quantifies the actual usability of colorized images from a task perspective, thereby achieving consistency between colorization quality evaluation and downstream task performance. To enhance applicability in complex application scenarios, RDS incorporates three key characteristics in its design: by utilizing an object matching mechanism based on Intersection over Union (IoU) [37], it reduces reliance on pixel-level registration and ensures evaluation stability under conditions where position errors exist, thereby possessing position robustness; by supporting both global performance assessment and category-level performance decomposition, the metric can not only reflect overall quality but also reveal performance differences in the model across different object categories, providing fine-grained interpretability; and by combining with a category division strategy, the evaluation dimension can be flexibly switched between color restoration capability and structure preservation capability according to application needs, thereby possessing task adjustability.

## 2. Methods

**Figure 1:**
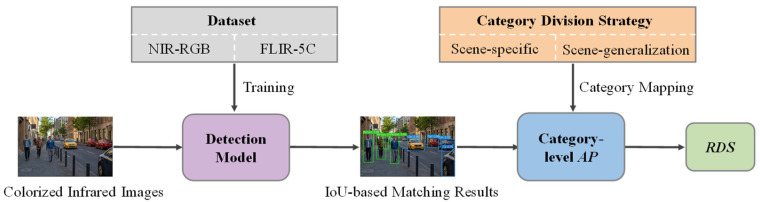
Overall computation pipeline of the RDS.

To achieve task-oriented colorization quality evaluation, this paper proposes the RDS metric. As illustrated in Figure 1, the pipeline begins with the construction of two experimental datasets (NIR–RGB and FLIR-5C), which provide real color images and corresponding annotations for detection model training. The trained model then serves as the proxy evaluator: colorized infrared images to be evaluated are fed into the detector, and predicted bounding boxes are matched against ground-truth annotations via an IoU-based mechanism, endowing RDS with position robustness. Category-level AP values are subsequently computed, providing fine-grained interpretability, and aggregated through category-weighted aggregation to yield the final RDS. A category division strategy is applied prior to AP computation to flexibly switch the evaluation focus between scene-specific mode and scene generalization mode, enabling task adjustability. The metric definition and computation pipeline are elaborated in Section 2.1; the category division strategy is detailed in Section 2.2; the dataset construction is described in Section 2.3; and the detection model training strategy is presented in Section 2.4.

### 2.1. RDS Metric

The ultimate goal of infrared image colorization lies not in pixel-level color restoration, but rather in enhancing downstream task performance of the colorized images. Traditional metrics measure images based on pixel differences or feature distributions, failing to reflect the preservation of semantic structure in images. Based on this, we design the RDS as a task-oriented evaluation metric. The design principle is: inputting the infrared colorized images to be evaluated into a trained object detection model, where the detection accuracy can measure the degree of semantic structure preservation and task usability of the infrared colorized images, serving as a proxy evaluation indicator for colorization quality.

The computation process of RDS includes the following three steps:

(1) Train object detection model.

This paper selects YOLOv5s [38] as the detection network, which, as a classic single-stage lightweight architecture, balances high detection accuracy with low computational complexity, making it suitable for deployment in large-scale colorized image evaluation scenarios. YOLOv5s is trained on a dataset constructed from real color images and their corresponding object annotations until the model achieves high detection accuracy. Since the category definition of the detection task can be adjusted according to the actual requirements, the RDS metric possesses task adjustability.

(2) Detect colorized images.

The infrared colorized images to be evaluated are input into the trained detection model to obtain predicted bounding boxes, category labels, and confidence scores for each image. Subsequently, these are matched with reference annotation boxes, and following the standard detection evaluation process, the Average Precision (AP) is calculated for each category. This process not only quantifies the semantic preservation of colorized images in detection tasks but also provides fine-grained information on the model’s performance across different object categories, enabling the RDS to possess fine-grained interpretability.

(3) Calculate RDS.

Based on the AP results of each category, RDS obtains an overall score through category-weighted aggregation. By default, this paper defines the final RDS metric in the form of mean value, namely:

(1) $$RDS=\frac{1}{N}\sum_{i=1}^{N}AP_{i}$$

where *N* represents the total number of detection categories, and *i* is the category index. To enhance robustness, RDS adopts an IoU-based matching mechanism when matching predicted and annotated boxes, with the judgment threshold, *τ*, set to 0.5. This strategy can tolerate minor position errors, effectively mitigating interference from insufficient pixel-level registration, enabling the RDS to possess position robustness.

In terms of task selection, the RDS adopts object detection as the downstream task for colorized images, considering that the primary purpose of infrared image colorization is to address its limitations in color space degrees of freedom and insufficient semantic expression capability. Compared to image-level tasks such as image classification, object detection not only requires the model to recognize object categories in images but also needs to localize their spatial boundaries, making it more sensitive to local color, texture, and structural information in images. This attribute makes it an ideal choice for evaluating whether colorization results effectively enhance semantic expression capability. In addition, the output of detection tasks possesses category decomposition capability and spatial matching mechanism, accommodating RDS’s design requirements for fine-grained interpretability and position robustness. By using detection accuracy as the evaluation criterion, RDS can measure the support capability of colorized images for downstream applications from a task-oriented perspective, thereby providing more informative feedback for model optimization and scenario adaptation.

### 2.2. Category Division Strategy

**Figure 2:**
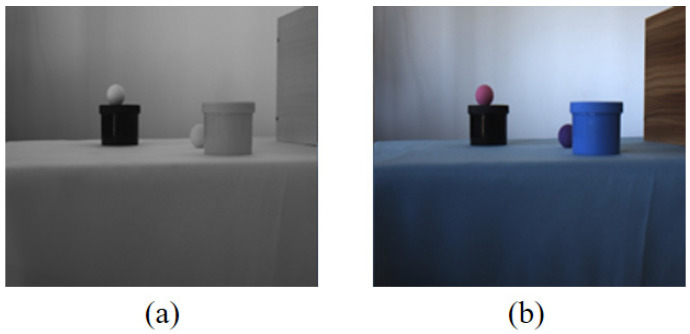
Example of single grayscale value corresponding to multiple color values. (**a**) Near-infrared grayscale image; (**b**) visible light grayscale image.

Due to the limited grayscale value range of infrared grayscale images and the lack of strong correlation between grayscale and object colors, the colorization process inevitably encounters the single-shape-multiple-color problem, where the same grayscale shape corresponds to multiple possible colors. For example, Figure 2 shows a set of images from our dataset, where two spheres in the near-infrared image: (a) have similar grayscale values, but in the color reference image and (b) one appears pink while the other appears purple. For observers familiar with this scene, colors can be inferred based on memory, but in unseen scenes, color prediction exhibits multi-solution characteristics, meaning that even if the generated colors differ from the reference, they may still be reasonable results. This ambiguity has a direct implication for evaluation: in familiar scenes where color information can be learned and memorized, the metric should emphasize the color restoration capability; in unseen scenes where color prediction is uncertain, the metric should instead emphasize the structure preservation capability to avoid penalizing reasonable colorization results. Therefore, category division must be flexibly adjusted according to the test scenario to achieve task-consistent evaluation criteria. The two category mapping modes proposed below are designed precisely to operationalize this distinction.

Let the original category set be:

(2) $$C_{orig}=\left\{\left(s_{i},c_{i}\right)\left|s_{i}\in S,c_{i}\in C\right.\right\},$$

where *S* represents the shape set, *C* represents the color set, and [symbol] represents categories jointly defined by shape, *s_i_*, and color, *c_i_*. This paper proposes two category mapping modes:

(1) Scene-specific mode.

When test images come from familiar (identical or similar) scenes as the training set, both shape and color dimensions are retained, namely:

(3) $$C_{memory}=C_{orig}.$$

This mode can directly evaluate the colorization model’s learning and memory capability for color details in familiar scenes, making RDS more sensitive to color prediction and suitable for analyzing the model’s color restoration performance.

(2) Scene generalization mode.

When test images come from unseen scenes, color prediction exhibits strong uncertainty, and overemphasizing color consistency leads to evaluation bias. Therefore, the color dimension is ignored, retaining only the shape attribute:

(4) $$C_{general}=\left\{s_{i}\left|s_{i}\in S\right.\right\}.$$

This mode focuses on evaluating whether the model maintains semantic structural information, avoiding unreasonable penalties due to color multi-solution characteristics, which better aligns with the goals of evaluating generalization performance.

To ensure the objectivity and reproducibility of the RDS across different studies, we provide the following guidelines for category mapping decisions:

(1) Determine the applicable mode. If test images are drawn from scenes seen during training, apply scene-specific mode, retaining full category definitions, including color attributes. If test images come from entirely new scenes that are not present in the training set, apply the scene generalization mode.
(2) Identify merge groups under the scene generalization mode. Within the original category set, subcategories that share the same shape attribute but differ only in color attribute (i.e., categories subject to the single-shape-multiple-color problem) are candidates for merging. Merging should be applied when the grayscale values of these subcategories overlap substantially in the infrared domain, confirming that their color distinction is not reliably inferable from the infrared input signal.
(3) Report the applied strategy. All RDSs should be accompanied by a clear statement of the mode applied, the resulting category mapping, and the criterion used to determine the merge groups to ensure cross-study comparability.

By flexibly switching between the scene-specific mode and scene generalization mode, the RDS can adjust evaluation priorities according to actual application requirements: emphasizing color restoration in seen scenes and highlighting structure preservation in unseen scenes. This flexible category definition approach enables the RDS to no longer depend on fixed evaluation dimensions, but rather dynamically adjust judgment criteria according to task objectives, thereby possessing good task adjustability.

### 2.3. Datasets

To ensure diversity in evaluation metric validation and comprehensiveness of results, this paper constructs and utilizes two types of experimental datasets: the near-infrared-to-color image pair dataset (NIR–RGB dataset) and the FLIR-5C dataset, which are used to simulate indoor and outdoor scenes, respectively. Among them, the NIR–RGB dataset is collected by this paper, covering multiple typical objects and background combinations, featuring controlled shooting conditions and high registration accuracy between near-infrared and color images, which helps evaluate the model’s learning and memory capability for color details under ideal imaging conditions. The FLIR-5C dataset is constructed based on the publicly available FLIR [39] thermal dataset through category filtering and grayscale processing, containing diverse traffic scenes and five object categories, with complex backgrounds and wide target distribution, suitable for analyzing the model’s generalization performance in real-world scenes. Both datasets are divided into the training set, Seen Test Set, and Unseen Test Set. Images in the Seen Test Set come from the same or similar scenes as the training set, while the Unseen Test Set consists of completely independent new scenes.

#### 2.3.1. NIR–RGB Dataset

This dataset aims to construct indoor near-infrared-to-color image pairs with high registration accuracy and introduce diversified backgrounds to support systematic evaluation. To this end, this paper employs a multispectral area-scan camera FSFE-1600D-10GE, which uses prism light combination to collect near-infrared and color image data, solving the image pair position misalignment problem at the hardware level.

**Figure 3:**
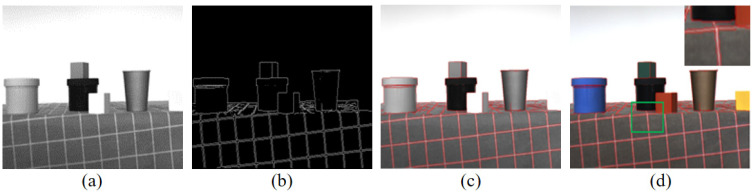
Edge alignment schematic diagram. (**a**) Near-infrared grayscale image; (**b**) edge feature map of near-infrared grayscale image; (**c**) (**a**) overlaid with (**b**); (**d**) visible light color image overlaid with (**b**).

Figure 3 shows the position-matching situation when the collected image pairs are downsampled to the 640 × 480 resolution. From Figure 3d and the enlarged area within the green box, it can be seen that the data collected by this camera has a very high degree of position matching, meeting the requirements of this paper’s experiments.

**Figure 4:**
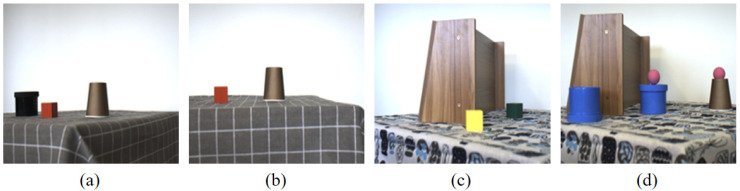
NIR–RGB dataset composition schematic diagram. (**a**) Image from Scene 1; (**b**) image from Scene 1; (**c**) image from Scene 2; (**d**) image from Scene 3.

In terms of scene design, this paper selects 6 types of typical objects as recognition targets: black jar, red cube, brown cup, white cup, pink ball, and purple ball. These targets are placed against backgrounds composed of tablecloths with different patterns and shelves in different arrangements, forming diverse scenes. As shown in Figure 4, (a) and (b) are images captured from different angles of scenes composed of black jar, red cube, and brown cup targets against a gray checkered tablecloth background; (c) and (d) are images captured from different scenes composed of different target combinations against backgrounds of white patterned tablecloth and side-standing shelves.

The NIR–RGB dataset contains approximately 2000 pairs of near-infrared-to-color images, of which approximately one-tenth of the independent scene images are divided into the Unseen Test Set, with these scenes not appearing at all in the training set; the remaining scenes are randomly divided into training set and Seen Test Set at a 4:1 ratio. Table 1 provides data statistics for each division.

#### 2.3.2. FLIR-5C Dataset

**Figure 5:**
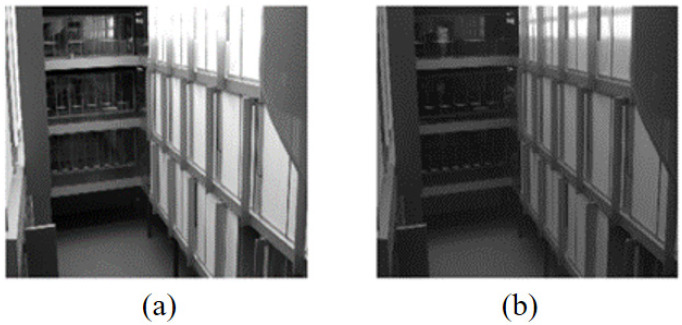
Comparison between near-infrared grayscale image and visible light grayscale image. (**a**) Near-infrared grayscale image; (**b**) visible light grayscale image.

Due to issues such as weak image stabilization performance, strong power supply dependency, and inconvenient equipment portability of multispectral cameras in outdoor environments, directly collecting high-quality outdoor near-infrared-to-color image pair datasets is very difficult. As shown in Figure 5, the structural features of grayscale-converted color images exhibit high similarity with actual near-infrared images, indicating that visible light grayscale images can reasonably approximate near-infrared images structurally. Therefore, this paper selects the publicly available FLIR thermal dataset as a basis and constructs the FLIR-5C dataset through category filtering and grayscale processing.

**Figure 6:**
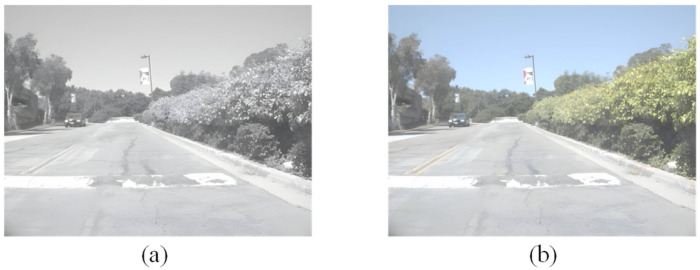
A pair of images from the FLIR-5C dataset. (**a**) Visible light grayscale image; (**b**) visible light color image.

The original FLIR dataset covers diverse traffic scenarios with comprehensive annotation information, containing over 10 target categories. To enhance the evaluation consistency and category distribution balance, this paper retains only samples containing five categories with abundant instances: person, car, bus, traffic light, and traffic sign. Images containing the above five categories are filtered as valid samples, and their color versions are converted to grayscale to construct grayscale-to-color image pairs, simulating a data environment where infrared and color images have good position matching in outdoor scenes. Figure 6 shows an example image pair from the FLIR-5C dataset. Based on the scene independence principle, 1033 images constitute the Unseen Test Set, ensuring that these scenes do not appear in the training set; the remaining 9346 images are divided into training set and Seen Test Set at a 9:1 ratio, used for colorization model training and familiar scene testing, respectively. Data division details are shown in Table 2.

### 2.4. Training Strategy

To obtain stable and reliable detection performance, this paper trains YOLOv5s-based detection models on the NIR–RGB and FLIR-5C datasets respectively. During training, all images are uniformly scaled to 640 × 480 resolution, and multi-scale data augmentation strategies (including image flipping and color perturbation) are adopted to improve model generalization capability. The number of training epochs is set to 100, the optimizer is Adam, and the initial learning rate is set to 1 × 10^−3^, and it gradually decreases to 1 × 10^−6^ using a cosine annealing strategy with epochs. Other parameters maintain YOLOv5s default configurations.

The following platform information is provided for reference to help readers estimate the computational cost of applying the RDS in practice. The experiments are completed on the Ubuntu operating system, with the training platform including: PyTorch 1.11.0 framework, Intel Xeon(R) Platinum 8255C processor, 43 GB memory, and NVIDIA RTX 3090 (24 GB VRAM) GPU.

## 3. Experiments

### 3.1. Experimental Setup

To verify the performance of the proposed RDS metric in practical colorization tasks, this paper designs a series of comparative experiments, covering the colorization model selection, evaluation metric settings, and test task configurations. By constructing colorization results with obvious quality differences and introducing multiple commonly used evaluation metrics for comparison, we systematically evaluate the effectiveness and advantages of RDS.

In terms of colorization models, three representative deep learning models are selected: CycleGAN, Pix2pix, and TIC-CGAN. These three models cover the spectrum from unconditional generative networks and conditional generative networks to improved models with structural constraints, providing sufficient differences in generation strategies and visual performance. The colorization models are not the focus of this paper; their main role is to construct colorization result samples with clearly distinguishable quality levels for metric validation. The selection follows three criteria: first, the three models are widely adopted baselines in the infrared colorization literature, ensuring that their relative performance characteristics are well-understood and externally verifiable; second, they produce colorization results with clearly distinguishable visual quality, which is a prerequisite for rigorously testing whether an evaluation metric possesses sufficient discriminative power; and third, they represent architecturally diverse approaches, reducing the risk that any observed metric behavior is specific to a particular network design. To ensure fairness of comparison and experimental reproducibility, these three models are trained for 500 epochs on the NIR–RGB dataset and 100 epochs on the FLIR-5C dataset to adapt to different data scales and task complexity. During training, all images are scaled to the 640 × 480 resolution. All models use the Adam optimizer, with the initial learning rate set to 1 × 10^−3^ and gradually decreasing to 1 × 10^−6^ using a cosine annealing strategy, with other training parameters maintaining the default settings of each method.

In terms of evaluation metrics, this paper selects three classic image quality evaluation methods as comparison baselines: SSIM, PSNR, and FID. These metrics are widely applied in image restoration and generation tasks and are capable of measuring image quality from perspectives of structural similarity, signal-to-noise ratio, and distribution distance. Through the comparison with these metrics, we can systematically evaluate the performance differences and advantages of the RDS in colorization tasks.

To keep the focus on metric evaluation rather than model comparison, the experiments in Section 3.2, Section 3.3, Section 3.4 and Section 3.5 are organized around the effectiveness of the RDS and its three key characteristics—position robustness, fine-grained interpretability, and task adjustability—with the three colorization models serving solely as sources of test samples with distinguishable quality levels. Specifically, this paper conducts analysis from four perspectives. First, through subjective–objective comparison experiments, we verify whether the RDS is consistent with human perception, thereby evaluating its effectiveness. Second, we introduce position perturbation to test the robustness of each metric, verifying its position robustness. Furthermore, we analyze the model’s performance differences across different targets through category-level detection accuracy, evaluating RDS’s fine-grained interpretability. Finally, combined with category division strategies, we compare RDS variations under scene-specific mode and scene generalization mode category definition modes, verifying its task adjustability.

### 3.2. Effectiveness Validation

To verify the effectiveness of the RDS under standard registration conditions, this section conducts analysis from both subjective perception and objective metrics perspectives, focusing on comparing the consistency between RDS and traditional metrics, as well as human evaluation results.

**Figure 7:**
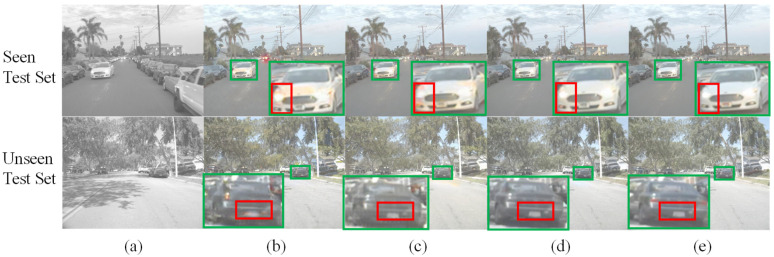
Test results on the FLIR-5C dataset. (**a**) Infrared grayscale image; (**b**) CycleGAN; (**c**) Pix2pix; (**d**) TIC-CGAN; (**e**) real color image. Green boxes indicate zoomed in regions, and red boxes indicate regions of key focus.

**Figure 8:**
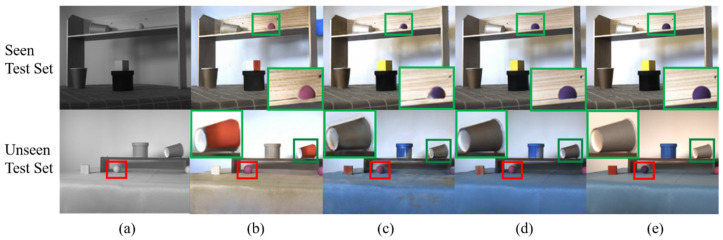
Test results on the NIR–RGB dataset. (**a**) Near-infrared grayscale image; (**b**) CycleGAN; (**c**) Pix2pix; (**d**) TIC-CGAN; (**e**) color image. Green boxes indicate zoomed in regions, and red boxes indicate regions of key focus.

Figure 7 and Figure 8 show typical colorization results on the FLIR-5C and NIR–RGB datasets. From the subjective visual effect perspective, images generated by TIC-CGAN have natural colors and well-preserved details, with the overall style closest to real color images; Pix2pix performs well in color consistency, but local details are blurred with artifacts appearing in edge regions; CycleGAN shows the most obvious color deviation, with lack of coordination between foreground and background, and the lowest detail fidelity. Overall, the subjective ranking for both datasets is TIC-CGAN > Pix2pix > CycleGAN.

To further verify the consistency of subjective judgment, Table 3 and Table 4 respectively list the average evaluation results of each model on the Seen Test Set and Unseen Test Set of the FLIR-5C and NIR–RGB datasets, including four metrics: SSIM, PSNR, FID, and the proposed RDS. The results show that under both datasets and both test subsets, all four metrics demonstrate consistent model ranking: TIC-CGAN scores highest, with TIC-CGAN > Pix2pix > CycleGAN. For example, on the FLIR-5C Unseen Test Set, the RDS of the three models are 39.87% (TIC-CGAN), 33.78% (Pix2pix), and 30.94% (CycleGAN), with the ranking completely consistent with subjective evaluation (i.e., the qualitative visual inspection of colorization results presented in Figure 7 and Figure 8).

In summary, RDS can accurately quantify quality variations in colorization results in standard registration scenarios, with its evaluation results not only highly consistent with subjective perception but also maintaining good consistency with existing metrics, verifying its effectiveness as an image colorization quality evaluation metric.

### 3.3. Position Robustness Validation

To verify the stability of RDS under registration error conditions, this section introduces artificial misalignment experiments to evaluate the performance changes in each metric before and after image misalignment. Given that infrared images in the FLIR-5C dataset are derived from grayscale conversion of visible light color images, the original image pairs are perfectly aligned at the pixel level, providing an ideal baseline for constructing artificial misalignment samples. Specifically, for each image, one direction (up, down, left, or right) is randomly selected, edge regions of 1~5 pixels width are cropped, and the image is restored to its original size through bilinear interpolation.

To quantify the performance changes in different metrics before and after misalignment, this paper introduces “Score Gap” as an auxiliary analysis indicator, defined as the absolute difference between the best and worst model scores in the evaluation results. The larger the Score Gap, the stronger the metric’s ability to distinguish model quality; the greater the decrease in Score Gap, the worse the metric’s stability under perturbation.

**Figure 9:**
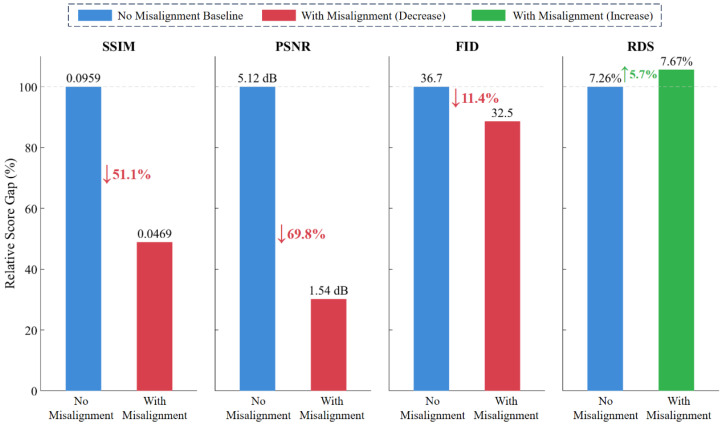
Comparison of Score Gap changes in each evaluation metric on FLIR-5C dataset Seen Test Set before and after misalignment. The vertical axis represents the relative Score Gap percentage with the no-misalignment Score Gap as the 100% baseline, and the values at the top of bars indicate the original Score Gaps. Blue bars represent the no-misalignment baseline, red/green bars represent the relative Score Gaps after misalignment, with arrows indicating change rates.

Figure 9 shows the Score Gap changes in each metric on the FLIR-5C dataset Seen Test Set before and after misalignment. As can be observed from the figure, all three traditional metrics—SSIM, PSNR, and FID—exhibit significant degradation after misalignment. Among them, the Score Gap of PSNR decreases from 5.12 dB to 1.54 dB, with a reduction rate as high as 69.8%, indicating its strongest dependence on pixel-level alignment; the Score Gap of SSIM decreases from 0.0959 to 0.0469, with a reduction rate of 51.1%; the Score Gap of FID decreases from 36.7 to 32.5, with a reduction rate of 11.4%. These results demonstrate that traditional metrics significantly decline in their ability to distinguish model quality under registration error scenarios.

In contrast, the Score Gap of the RDS remains essentially stable, changing marginally from 7.26% to 7.67%; this negligible difference falls within normal statistical fluctuation of AP computation over a finite test set and does not indicate a directional improvement, but rather confirms that RDS maintains stable discriminability under position perturbation. This characteristic stems from RDS’s adoption of an IoU-based object matching mechanism during the calculation process, making it insensitive to pixel-level position shifts, thereby possessing good position robustness and enhancing reliability in practical applications.

### 3.4. Fine-Grained Interpretability Validation

**Figure 10:**
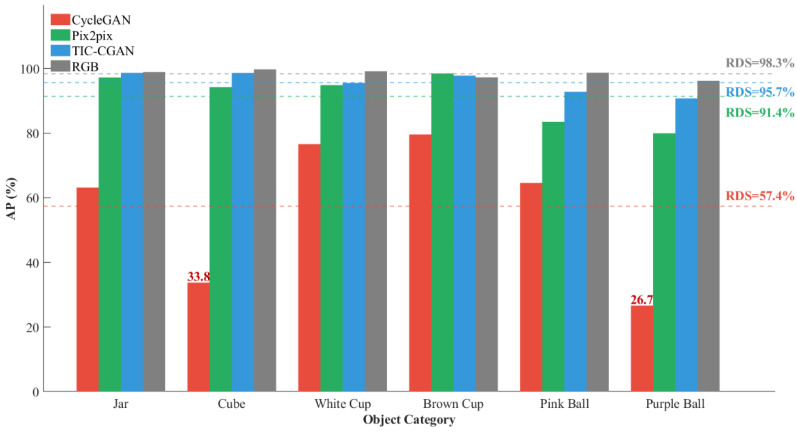
Category-level average evaluation results and RDS visualization analysis of each model on NIR–RGB Seen Test Set.

To verify the fine-grained interpretability of the RDS, this section analyzes its category-level scoring results. Figure 10 shows the AP values of each method across six detection categories on the NIR–RGB dataset Seen Test Set, where the detection results of real RGB images (RDS = 98.3%) serve as the performance upper bound. The RDSs of each model are marked with dashed lines to facilitate the observation of the relationship between average performance and category-level performance.

Using real RGB images as the reference baseline, TIC-CGAN achieves AP values of 98.7% and 98.7% on the “jar” and “cube” categories, respectively, almost identical to the RGB baseline (98.9%, 99.7%), indicating that this model possesses strong structural preservation capability on shape-dominant targets. For color-dominant categories such as “white cup,” “brown cup,” “pink ball,” and “purple ball,” TIC-CGAN also maintains AP values above 90%, with an overall RDS of 95.7%, only 2.6 percentage points away from the RGB baseline, demonstrating its strong scene memory and color restoration capability.

Further comparing Pix2pix with TIC-CGAN, it can be concluded that both perform similarly on shape-dominant categories, but show AP gaps of approximately 9% and 11% on the “pink ball” and “purple ball” categories, respectively. This difference corresponds to the phenomenon in the first row of Figure 8, where Pix2pix shows color restoration deviations on spheres, indicating that Pix2pix still has deficiencies in learning color details, resulting in an overall RDS of 91.4%.

CycleGAN’s category-level performance shows obvious imbalance characteristics. For the “white cup” and “brown cup” categories, its AP can still reach 76.6% and 79.6%, but for the “cube” and “purple ball” categories, the AP values are only 33.8% and 26.7%, respectively, far lower than other models. These two severe shortcomings directly pull down its overall RDS to 57.4%. The above quantitative results are consistent with the distortion and discoloration phenomena in CycleGAN output images in Figure 8, verifying the reliability of RDS category-level analysis results.

In summary, RDS can reveal model performance differences across different targets through category-level AP decomposition. It can not only identify the average performance level of models but also precisely locate their weaknesses in specific categories, providing clear basis for performance diagnosis and targeted optimization, possessing fine-grained interpretability that traditional metrics (SSIM, PSNR, FID) do not have.

### 3.5. Task Adjustability Validation

To verify the RDS’s adaptability under different task objectives, this paper designs task adjustability validation experiments on the Unseen Test Set of NIR–RGB, examining RDS’s evaluation capability under two task settings: color restoration-oriented and structure preservation-oriented.

The experiment employs two category definition strategies: the scene-specific mode (six-category setting) subdivides targets into six categories of jar, cube, white cup, brown cup, pink ball, and purple ball, emphasizing learning and restoration capability for color details; the scene generalization mode (4-category setting) merges targets by shape dimension into four categories of jar, cube, cup, and ball, reducing dependence on color consistency and focusing more on structure and semantic information.

Table 5 shows the RDSs and category-level AP values of the three colorization models under these two category settings. From the table, it can be observed that under the six-category setting, TIC-CGAN’s AP on “purple ball” is only 22.00%, significantly pulling down the overall score to 76.05%. This is consistent with the phenomenon in Figure 8, where the model incorrectly colors the purple ball as pink. Due to the influence of multi-solution characteristics of colors, forcibly distinguishing the color dimension in unseen scenes easily leads to evaluation distortion.

Under the four-category setting, pink ball and purple ball are unified as the “ball” category, eliminating the above penalty. TIC-CGAN’s AP increases to 100%, and the overall RDS also rises to 96.45%. Other models show similar trends, indicating that under scene generalization conditions, adjusting category definitions can effectively avoid misjudgment of reasonable coloring results, making evaluation results more consistent with human perception and application objectives.

In summary, through switchable category mapping methods, RDS enables the evaluation dimension of colorization models to shift from color restoration capability to structure preservation capability, achieving flexible adjustment of evaluation dimensions under task-oriented guidance, demonstrating good task adjustability.

## 4. Conclusions

This paper proposes a task-oriented colorization image evaluation metric RDS, which achieves consistency between colorization quality evaluation and downstream task performance by using the recognition accuracy of object detection models on colorized images as a proxy evaluation indicator for image quality. This paper designs four types of experimental tasks to evaluate the effectiveness and advantages of the RDS. Results demonstrate that the RDS is not only highly consistent with subjective perception but also maintains good consistency with existing objective metrics; maintains stable discriminability in position perturbation scenarios, verifying its position robustness; supports category-level performance decomposition, revealing model performance differences across different object categories and demonstrating fine-grained interpretability; and when combined with category division strategies, RDS can switch evaluation dimensions between color restoration and structure preservation according to task objectives, exhibiting good task adjustability. Quantitatively, its discriminative Score Gap improves by 5.7% under registration error scenarios while PSNR, SSIM, and FID degrade by up to 69.8%, 51.1%, and 11.4%, respectively; category-level AP decomposition exposes per-category weaknesses—such as CycleGAN’s AP of only 26.7% on the “purple ball” category—that are completely hidden by global scores; and switching from scene-specific mode to scene generalization mode reduces evaluation distortion in unseen scenes, raising TIC-CGAN’s RDS from 76.05% to 96.45%. Overall, the RDS provides a new evaluation metric with enhanced usability and stability for colorization results.

Future work can further apply RDS to broader generation and translation tasks such as night vision enhancement and remote sensing false color, improving its applicability across different data types and application scenarios. Meanwhile, it can also be combined with more complex downstream tasks such as semantic segmentation and action recognition to further expand the coverage scope of task-oriented metrics. In addition, validating the consistency of RDSs across different proxy detection architectures—such as YOLOv8 and Faster R-CNN—represents an important next step to further establish the metric’s generalizability, and it is planned as a priority item in our subsequent work.

Furthermore, applying RDS to evaluate state-of-the-art colorization methods based on Diffusion Models and Vision Transformers would provide a broader benchmark and further demonstrate the metric’s discriminative power across the modern research landscape, representing a natural and straightforward extension of the current work. A formal quantitative correlation analysis between RDSs and human subjective ratings—such as Spearman’s Rank Correlation Coefficient computed over a larger set of colorization methods—would further characterize the metric’s perceptual alignment and is planned as part of a dedicated follow-up study.

## Figures and Tables

**Table 1:** NIR–RGB dataset detailed information.

Data Type	Number of Image Pairs	Number of Targets	Number of Scenes
Training set	1443	4134	138
Seen Test Set	361	1025	
Unseen Test Set	205	651	33

**Table 2:** FLIR-5C dataset detailed information.

Data Type	Number of Image Pairs	Number of Targets	Number of Scenes
Training set	8403	129,172	132
Seen Test Set	943	14,742	
Unseen Test Set	1033	15,565	17

**Table 3:** Average evaluation results of each model on the FLIR-5C test set.

Dataset		Seen Test Set				Unseen Test Set			
	Metric	SSIM	PSNR/dB	FID	RDS	SSIM	PSNR/dB	FID	RDS
**Model**									
CycleGAN		0.8480	26.19	75.35	31.13%	0.8331	25.73	77.40	30.94%
Pix2pix		0.8994	28.81	57.82	34.60%	0.8805	27.73	66.16	33.78%
TIC-CGAN		0.9439	31.31	38.70	38.39%	0.9374	30.84	40.80	39.87%

**Table 4:** Average evaluation results of each model on the NIR–RGB test set.

Dataset		Seen Test Set				Unseen Test Set			
	Metric	SSIM	PSNR/dB	FID	RDS	SSIM	PSNR/dB	FID	RDS
Model									
CycleGAN		0.7878	17.01	136.2	57.39%	0.7153	14.89	146.2	50.81%
Pix2pix		0.8924	26.03	71.97	91.38%	0.7844	17.55	125.0	74.06%
TIC-CGAN		0.9109	25.79	31.26	95.71%	0.8162	18.08	74.35	76.05%

**Table 5:** Average evaluation results of each model on NIR–RGB Unseen Test Set under different category strategies.

Category Strategy		Scene-Specific Mode							Scene Generalization Mode				
Metric		AP						RDS	AP				RDS
	Target	Jar	Cube	White Cup	Brown Cup	Pink Ball	Purple Ball		Jar	Cube	Cup	Ball	
Model													
Cycle-GAN		74.04%	20.07%	75.87%	66.56%	62.45%	5.87%	50.81%	75.55%	34.49%	92.82%	90.68%	73.39%
Pix2pix		94.07%	85.52%	82.17%	73.91%	73.65%	35.04%	74.06%	93.41%	85.36%	96.99%	99.05%	93.70%
TIC-CGAN		96.12%	92.33%	86.34%	84.05%	75.46%	22.00%	76.05%	94.15%	94.53%	97.13%	100.0%	96.45%

## Data Availability

Data underlying the results presented in this paper are not publicly available at this time but may be obtained from the authors upon reasonable request. The FLIR-5C dataset is derived from the publicly available FLIR thermal dataset available at https://oem.flir.com/zh-cn/solutions/automotive/adas-dataset-form/ (accessed on 27 January 2026).
